# Initial investigation on ultrasound-guided percutaneous biopsy of lesions in the first hepatic hilum with fusion of ultrasound and multimodal imaging cognitive guidance

**DOI:** 10.3389/fonc.2024.1297153

**Published:** 2024-04-24

**Authors:** Xian-Tao Zeng, Xia Liang, Zhi-Liang Hong, Sheng Chen, Jian-Chuan Yang, Yu-cheng Lin, Song-Song Wu

**Affiliations:** ^1^ Shengli Clinical Medical College of Fujian Medical University, Fuzhou, China; ^2^ Fujian Provincial Key Laboratory of Critical Care Medicine, Fuzhou, China; ^3^ Department of Ultrasound, Fujian Provincial Hospital, Fuzhou, China; ^4^ Department of Ultrasonography, Affiliated Fuzhou First Hospital of Fujian Medical University, Fuzhou, China

**Keywords:** multi-modal imaging, cognitive fusion, first hepatic hilum, ultrasound-guided, core needle biopsy

## Abstract

**Purpose:**

This study aims to evaluate the efficacy and safety of ultrasound-guided percutaneous biopsy of the first hepatic hilum lesion, and examine its clinical value of diagnosis and treatment.

**Methods:**

We conducted a retrospective study on patients diagnosed with the first hepatic hilum lesions at Fujian Provincial Hospital between February 2015 and October 2022. We selected patients who had lesions in the first hepatic hilum(including a 2cm surrounding area of the left/right hepatic ducts and upper-middle segment of the common bile duct) and the liver periphery(in the peripheral area of the liver, outside of the above-mentioned first hepatic porta region). These patients underwent percutaneous ultrasound-guided core needle biopsy (PUS-CNB) with cognitive fusion guidance using CT, MRI, or PET-CT. We compared the safety and efficacy of PUS-CNB in the first hepatic hilum and the liver periphery to explore the value of PUS-CNB in optimizing the clinical treatment of the first hepatic hilum lesions.

**Results:**

The studied includes 38 cases of the first hepatic hilum cases (18 females; 20 males), 23 presented with mass-forming tumors while the remaining 15 exhibited diffuse infiltrative tumors, with an average diameter of 4.65± 2.51 cm. The percutaneous biopsy procedure, conducted under ultrasound guidance, had an average operation time of 14.55 ± 2.73 minutes, and resulted in a postoperative bleeding volume of approximately 10.79 ± 2.79 ml. The diagnostic success rate was noted to be as high as 92.11% among the participants who underwent percutaneous biopsy of the first hepatic hilum. Procedural complications, such as bleeding, bile leakage, intestinal perforation, infection or needle tract seeding, did not occur during or after the biopsy procedure. Affected by biopsy results, 5 altered their clinical treatment plans accordingly, 24patients received non-surgical treatment, 9 underwent surgical treatment, 5 underwent radiofrequency ablation for the lesions. The study comprised a total of 112 cases for percutaneous biopsy of the liver periphery. The safety and effectiveness of the two biopsy techniques were comparable, with diagnostic success rates of 92.11% VS. 94.34%, respectively (***p*** = 0.61).

**Conclusion:**

Cognitive fusion of ultrasound and multi-modal imaging for the first hepatic hilum lesion puncture biopsy is a safe and effective diagnostic procedure, with better diagnostic rate, may improve clinical value of diagnosis and treatment of various diseases.

## 1 Introduction

The first hepatic hilum, located on the visceral surface of the liver, is a complex anatomical region where multiple structures converge, including the portal vein, hepatic artery, bile duct, lymphatics, nerves, and connective tissue. Lesions in this area pose a diagnostic challenge as imaging features of benign and malignant tumors can overlap, resulting in approximately 13-15% of preoperatively suspected malignancies being diagnosed as benign (1, 2). Clinicians should be aware of these challenges when evaluating patients with suspected lesions in the first hepatic hilum. Surgical resection of lesions located in the first hepatic hilum represents a formidable challenge, with reported rates of severe complications ranging from 37-64%, and surgical mortality rates of 8-10% (3). As such, obtaining an accurate histological diagnosis prior to surgical intervention is essential for ensuring proper diagnosis and appropriate surgical management. This crucial step can help mitigate the risk of adverse outcomes and improve patient outcomes (4). The most commonly used biopsy techniques for diagnosing suspected malignancies in the bile duct include cytology using brush cytology during endoscopic retrograde cholangiopancreatography (ERCP) (5), forceps biopsy, and endoscopic ultrasound-guided fine-needle aspiration (EUS-FNA). However, ERCP is primarily used to sample the bile ducts and has a detection rate of 44% to 80% for suspected cholangiocarcinoma in that area (6, 7). The malignant tumor detection rate for forceps biopsy is 43% to 81% (8, 9). EUS-FNA demonstrates slightly higher diagnostic sensitivity (45%-86%) (10, 11). But lower negative predictive value (9%-38%) compared to previous techniques for obtaining pathological tissue of the first hepatic hilum lesions (12, 13). False negatives may occur, highlighting the need for alternative approaches to safely and effectively diagnose advanced malignant tumors and obtain tissue for genetic testing to facilitate targeted and immunotherapy.

Hence, there is an urgent need for novel strategies that can guarantee the safe and effective acquisition of pathological tissue from lesions in the first hepatic hilum, thereby enabling precise preoperative diagnosis. We conducts a study to explore a novel and safe percutaneous biopsy technique for detecting the first hepatic hilum lesions. The primary objective is to bolster diagnostic precision, furnish valuable insights for clinical deliberation, and promote better treatment selection, particularly in the context of malignant tumors.

## 2 Materials and methods

### 2.1 The subjects of study

**Figure 1:**
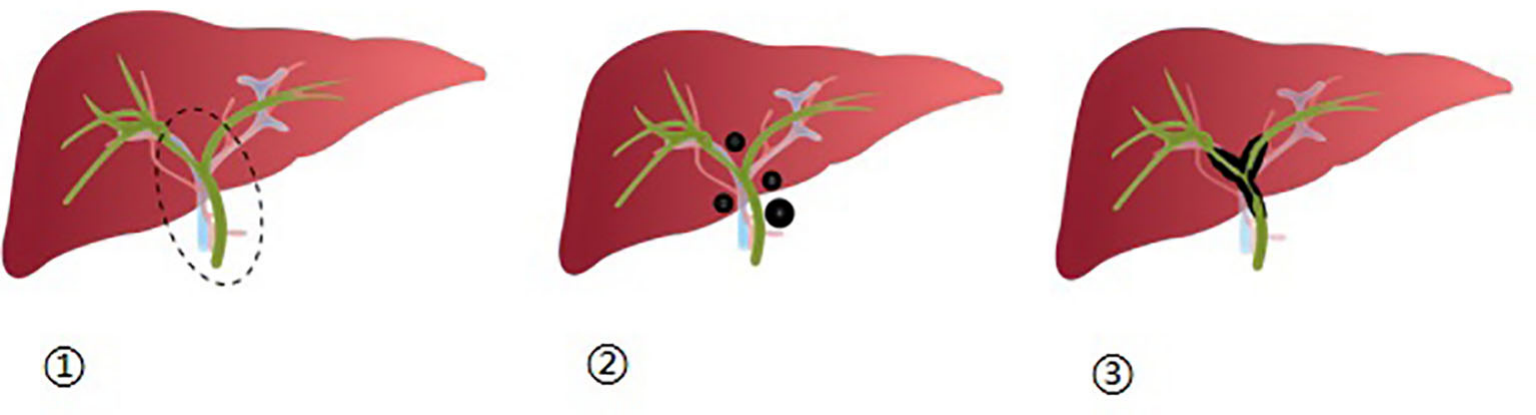
① Depicts the first hepatic hilum as a dashed elliptical area, which encompasses the surrounding 2 cm range of the left and right hepatic ducts, as well as the upper-middle region of the bile duct; ②: Illustrates the distribution of nodular lesions within the first hepatic hilum; ③: Displays the distribution of diffuse lesions within the first hepatic hilum.

**Figure 2:**
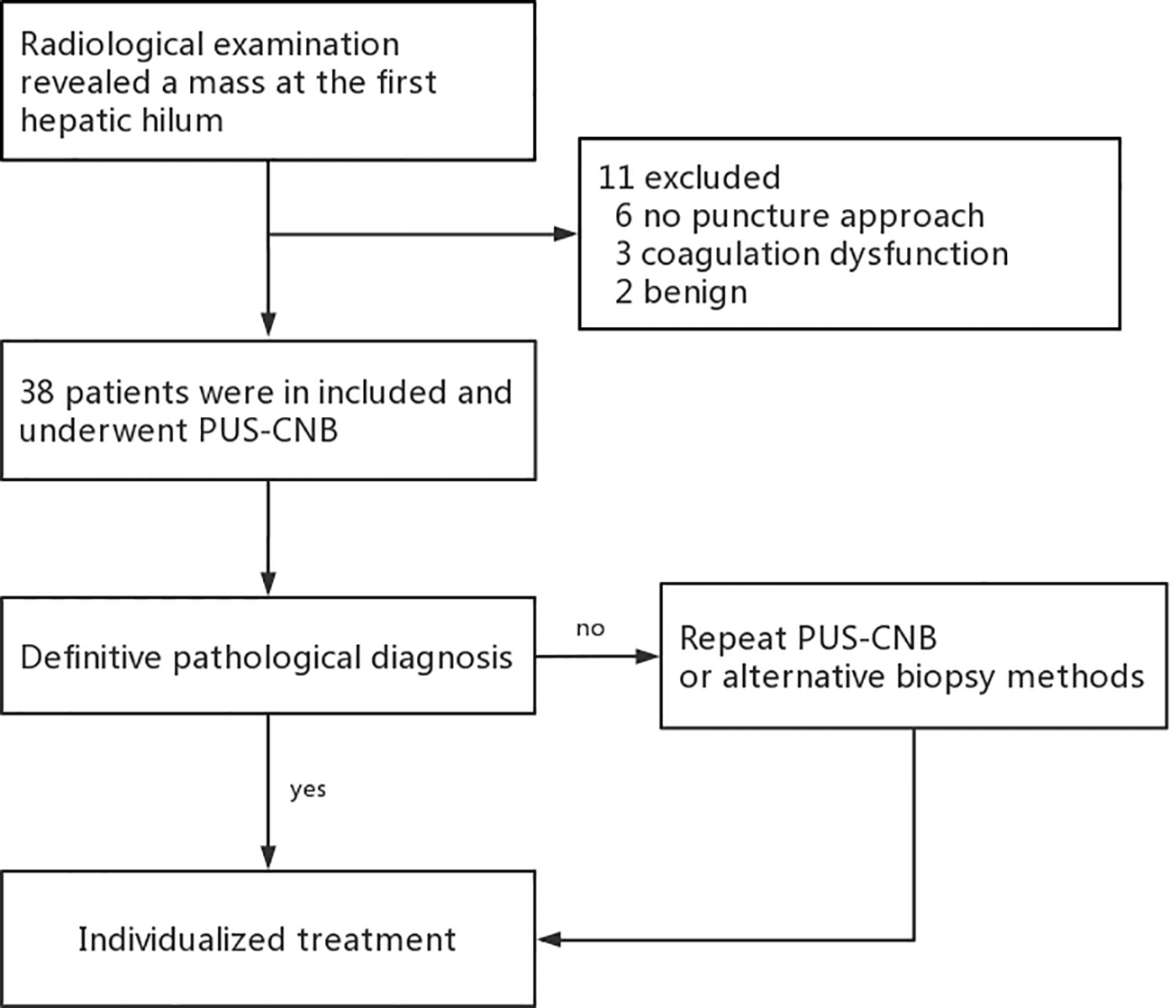
Flow chart.

This retrospective study encompassed cases between February 2015 and October 2022 at Fujian Provincial Hospital. The patients who were included in the study exhibited lesions within the first hepatic hilum(including a 2cm surrounding area of the left/right hepatic ducts and upper-middle segment of the common bile duct) (**Figure 1**) and the liver periphery(in the peripheral area of the liver, outside of the above-mentioned first hepatic porta region). Inclusion criteria:(a) Imaging findings indicating the first hepatic hilum or perihepatic masses requiring a definitive pathological diagnosis;(b) Availability of a safe and suitable transabdominal puncture route;(c) Platelet count >50×10^9/L;(d) Prothrombin time ratio ≥70%. Exclusion criteria:① Severe coagulation dysfunction;② Severe underlying diseases or physical weakness (**Figure 2**).

This retrospective analysis has been approved by the Institutional Review Board of Fujian Provincial Hospital. Written informed consent was obtained from each patient prior to treatment, with all patients being informed of the treatment indications, potential therapeutic effects, as well as possible complications that may arise.

### 2.2 Instruments and methods

In our clinical setting, We utilize the Philips iU22 and GE Vivid 7 Dimension color Doppler ultrasound diagnostic machines for our ultrasound equipment. The linear array probe operates at a frequency of 5-12MHz, while the convex array probe operates at a frequency of 2-5MHz. For our biopsy procedures, we employ either the 18G fully automatic biopsy gun or the semi-automatic option (BARD Magnum, MN18-20, CR).

### 2.3 Pre-puncture preparation

All patients underwent routine laboratory examinations, including complete blood counts, biochemical tests, and coagulation function assessments. Prior to the surgery, all patients underwent CT/MRI/PET-CT imaging to evaluate the first hepatic hilum and the surrounding areas using multimodal imaging. The surgical team performed a multimodal imaging assessment prior to the puncture procedure to reconstruct the specific location of the first hepatic hilum lesion and its surrounding anatomical relationships. The lesion was scanned using an ultrasound probe prior to the puncture, and then the imaging image in the mind was fused with the ultrasonic image to determine the puncture passage (a. intercostal approach above the rib arch, b. subcostal approach through the liver parenchyma).

### 2.4 Ultrasound-guided percutaneous biopsy procedure

The procedure is performed in the ultrasound intervention room: The patient was positioned supine, The ideal puncture site is identified through the process of cognitive fusion, using contrast-enhanced ultrasonography to visualize areas of abnormal enhancement (indicating an active lesion) and rapid washout (suggesting a suspicious region for malignant tumor). These findings guide the selection of the suspected active target area. The puncture site was sterilized and draped, and local anesthesia was administered to the area. Under real-time ultrasound guidance, the needle tip was observed to sequentially pass through the skin, subcutaneous tissue, and liver until reaching the lesion (taking care to avoid major blood vessels). The trigger (biopsy device) was activated to obtain tissue, and then the needle tip was withdrawn. All biopsies were performed under local anesthesia with 2% lidocaine. The biopsy procedure was carried out by two experienced ultrasound intervention physicians with over 10 years of experience. An 18G biopsy needle was used for the puncture, and after tissue sampling, the needle was quickly withdrawn. The quality of the tissue samples within the needle notch was observed, and if deemed unsatisfactory, additional samples were taken. Each time, 2-4 tissue samples measuring approximately 15mm-20mm in length were obtained for pathological diagnosis. After applying sterile dressing to the skin puncture site, patients were instructed to apply pressure to the site for 5 minutes and were observed for 30-60 minutes to check for any active bleeding. Additionally, patients were advised to rest in bed for 6 hours after the procedure, and no medication intervention was administered.

### 2.5 Evaluation indicators

**Diagnostic success rate assessment:** The diagnostic success rate was assessed as follows: Cases in which the biopsy pathology diagnosis was confirmed and matched the clinical discharge diagnosis or postoperative pathology diagnosis were considered successful cases. Cases in which the biopsy pathology diagnosis was confirmed but did not match the postoperative pathology diagnosis were considered non-matching cases. Cases in which the biopsy was unsuccessful and could not provide a definitive pathology diagnosis, requiring a repeat biopsy or surgical procedure for a definitive pathology diagnosis, were also considered non-matching cases. The diagnostic success rate was calculated as the number of cases with a confirmed pathology diagnosis on the first biopsy divided by the total number of cases.

**The effectiveness measure includes:** The average time spent during the operation.

**Safety indicators include (Complications of puncture):** Post-puncture, the amount of bleeding from the skin puncture site is evaluated. Following the surgery and after one hour, careful observation is conducted to assess the presence of fluid collections within the abdominal and pelvic cavities caused by active bleeding, as well as to check for any apparent damage to surrounding organs. The patients are monitored for symptoms of infection, bile leakage, intestinal perforation, and other related symptoms. During the follow-up period, which lasts for three months after the surgery, the presence of needle tract implantation metastasis and other long-term complications related to the procedure are observed using methods such as reviewing medical records, real-time communication with the attending physician, and conducting telephone follow-ups after the patient’s discharge.

### 2.6 Statistical processing

This study employed SPSS 26.0 statistical software and considered *p*<0.05 (two-tailed test) as the level of statistical significance. Independent sample t-tests and Mann-Whitney U tests were utilized for comparing quantitative data, while count data was represented using frequency with between-group differences assessed via chi-square tests and Fisher’s exact probability method.

## 3 Results

### 3.1 General information

In this study, a total of 49 patients with lesions in the first hepatic hilum were selected for percutaneous ultrasound-guided core needle biopsy (PUS-CNB). Among them, 11 cases were excluded due to the lack of a safe puncture pathway or abnormal coagulation function. The remaining 38 cases underwent PUS-CNB (20 males and 18 females) with a mean age of 61.08 ± 11.29 years (ranging from 30 to 77 years). The clinical symptoms upon admission included abdominal pain in 28 cases, jaundice in 15 cases, vomiting in 5 cases, constipation in 4 cases, and a less common symptom of recurrent melena in 1 case. The types of lesions included 23 cases of mass-type and 15 cases of diffuse infiltration, with an average diameter of 4.65 ± 2.51 cm. Prior to the puncture, 30 cases underwent MRI examination, 7 cases underwent CT examination, and 2 cases underwent PET-CT examination. Based on cognitive fusion guidance, the selected puncture approaches were intercostal transhepatic in 32 cases and subcostal transhepatic in 6 cases (See **Table 1**).

**Table 1:** General information for both groups.

index	first hepatic hilum	liver periphery
Total number of cases	38	112
Gender		
Male	20	66
Female	18	46
Age (years)		
Average	61.08 ± 11.29	58.79 ± 12.42
Focal type		
The mass type	23	94
diffuse type	15	18
Diameter of the lesion	4.65 ± 2.51	5.59 ± 3.85
puncture channel		
intercostal liver puncture approach	32	106
Subcostal space puncture approach	6	6

### 3.2 Safety and effectiveness of the puncture

The average duration of PUS-CNB procedure for the first hepatic hilum lesions was 14.55 ± 2.73 minutes. The postoperative bleeding volume was approximately 10.79 ± 2.79 ml. All patients experienced mild discomfort, and no complications such as bleeding, bile leakage, intestinal perforation, or infection were observed during or after the procedure. There were no evident needle tract metastases during the six-month follow-up. The success rate of tissue sampling through the biopsy procedure was 100%. The biopsy results revealed 19 cases of cholangiocarcinoma, 2 cases of gallbladder carcinoma, 5 cases of hepatocellular carcinoma, 1 case of diffuse large B-cell lymphoma, 4 cases of inflammatory lesions, 1 case of parasitic infection, 5 cases of metastatic tumors (non-hepatic or biliary in origin), and 1 case of malignant pheochromocytoma. Among them, three cases initially diagnosed as inflammatory lesions were confirmed as cholangiocarcinoma through surgery, resulting in a biopsy success rate of 92.11% (See **Tables 2**, **3**).

**Table 2:** Safety and efficacy comparison of the two groups.

index	first hepatic hilum	liver periphery	*t*	*p*
Total number of cases	38	112		
validity index				
puncture time(min)	14.55 ± 2.73	14.24 ± 3.01	0.59	0.56
Diagnostic success rate	92.11%	94.64%	/	0.57
Safety indicator				
bleeding Volume(ml)	10.79 ± 2.79	10.16 ± 2.30	1.25	0.22
infect	0	0		
Perforation	0	0		
Bile leakage	0	0		
needle track implantation	0	0		
postoperative hemorrhage	0	0		

**Table 3:** Pathological results of the two groups.

index	first hepatic hilum	liver periphery
cholangiocarcinoma	19	9
hepatocellular carcinoma	5	23
diffuse large B cell lymphoma	1	0
Inflammatory lesions	4	9
parasitic infection	1	0
metastatic tumor	5	69
Malignant pheochromocytoma	1	0
carcinoma of gallbladder	2	0
Neuroendocrine tumor	0	1
angioma	0	1
Total number of cases	38	112

### 3.3 Compared with perihepatic puncture biopsy

A total of 112 patients underwent ultrasound-guided percutaneous needle biopsy of the liver periphery with an average operation time of 14.24 ± 3.01 minutes and an average postoperative bleeding volume of approximately 10.16 ± 2.30 ml. All patients tolerated the procedure well, experiencing only mild pain, and no major complications such as significant bleeding, bile leakage, intestinal perforation, or infection were observed during or after the procedure. During the six-month follow-up, there were no evident needle tract metastases. The overall success rate of tissue sampling through the biopsy procedure was 94.64%, which did not show a significant difference compared to the success rate of the first hepatic hilum biopsies (P = 0.57). (See **Table 3**).

### 3.4 Post-puncture changes in diagnosis and treatment

After undergoing the first hepatic hilum biopsy, 5 patients had a change in their treatment plans (**Table 4**):

**Table 4:** Effect of PUS-CNB on the treatment.

Type	Effect of ultrasound-guided needle biopsy on therapy	Example number	Percentage of cases
ET1	Leading to inappropriate treatment options.	0	00.00
ET2	There was no effect on the choice of the treatment method	1	2.63
ET3	No change the choice of treatment but increased clinician confidence in the choice	27	71.05
ET4	Has an important role in the choice of treatment.	5	13.16
ET5	Change in the treatment approach chosen	5	13.16
	total	38	

ET, Effect on treatment.

**Figure 3:**
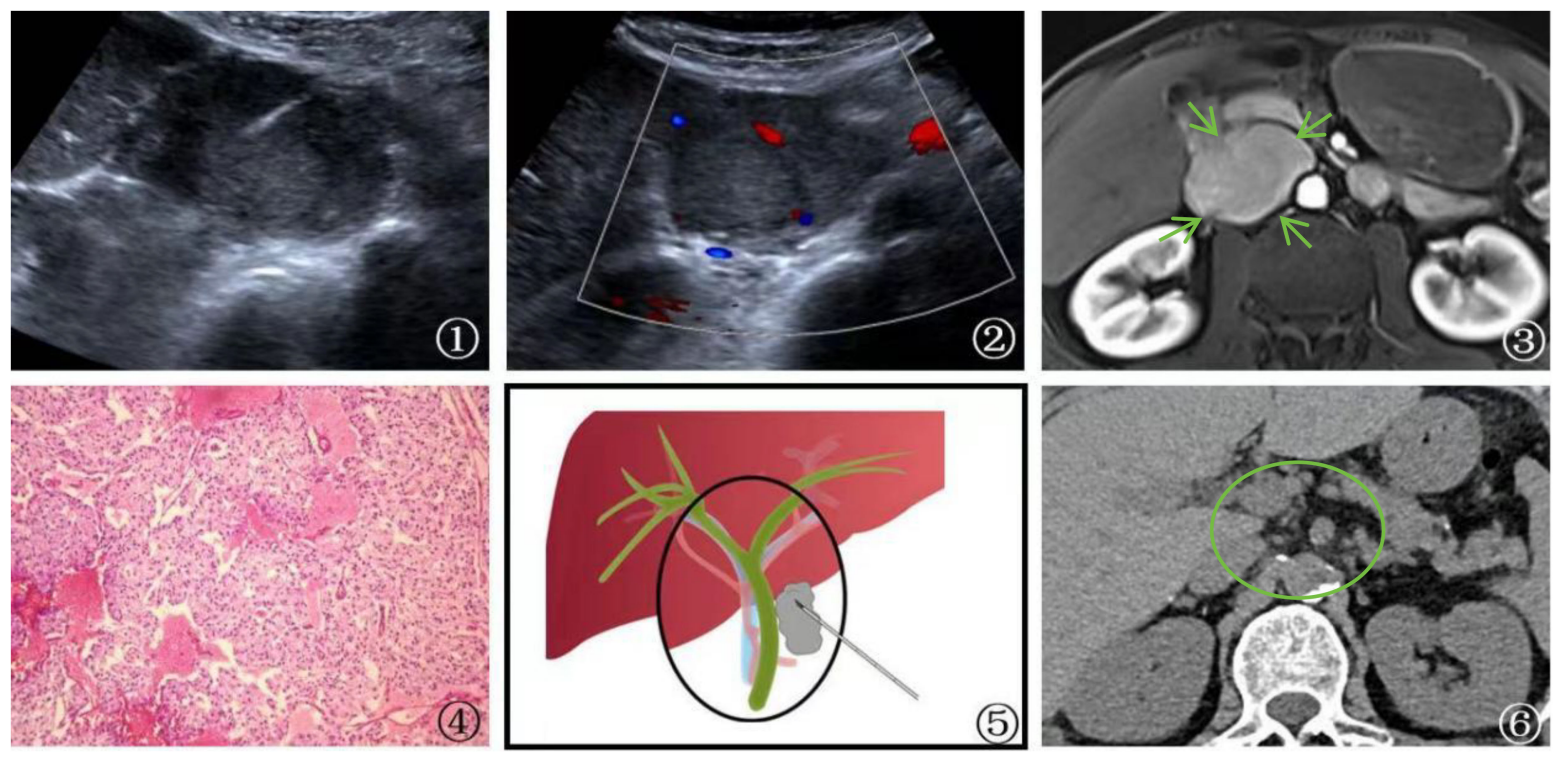
A 58-year-old male patient Presented with malignant pheochromocytoma. ①: Percutaneous biopsy of the first hepatic hilum was performed under ultrasound guidance. ②: Ultrasonography revealed a hyperechoic area in the first hepatic hilum with clear borders, regular shape, heterogeneous internal echogenicity, and slight blood flow signal. ③: MRI T1 arterial phase showed mild to moderate enhancement of the first hepatic hilum lesion(arrow), suggesting the possibility of metastatic tumor. ④: Schematic diagram of percutaneous biopsy of the first hepatic hilum under ultrasound guidance. ⑤: The neoplasm displays a fascicular and lobular architecture with active proliferative activity and local tissue invasion. Cystic degeneration is noted, and malignant tumor emboli are identified within the neoplastic thrombosed vessels and adjacent fibrovascular stroma. These features fulfill the criteria for malignant pheochromocytoma per the 2016 World Health Organization Classification of Neuroendocrine Tumors (magnification, ×10). ⑥: After 1 year of follow-up CT examination post-surgery, the lesion was completely excised (circle) and no evidence of recurrence was detected.

One patient with postoperative lung cancer was found to have the first hepatic hilum lesion on follow-up imaging, which raised suspicion of a possible metastatic tumor. However, PUS-CNB confirmed it as malignant pheochromocytoma, leading to a referral for urological treatment (**Figure 3**).

One patient with postoperative gastric cancer was found to have a first hepatic hilum lesion on follow-up imaging, along with a significant increase in AFP levels. Clinical considerations pointed to primary liver cancer, but PUS-CNB confirmed it as a metastatic carcinoma, resulting in chemotherapy treatment.

**Figure 4:**
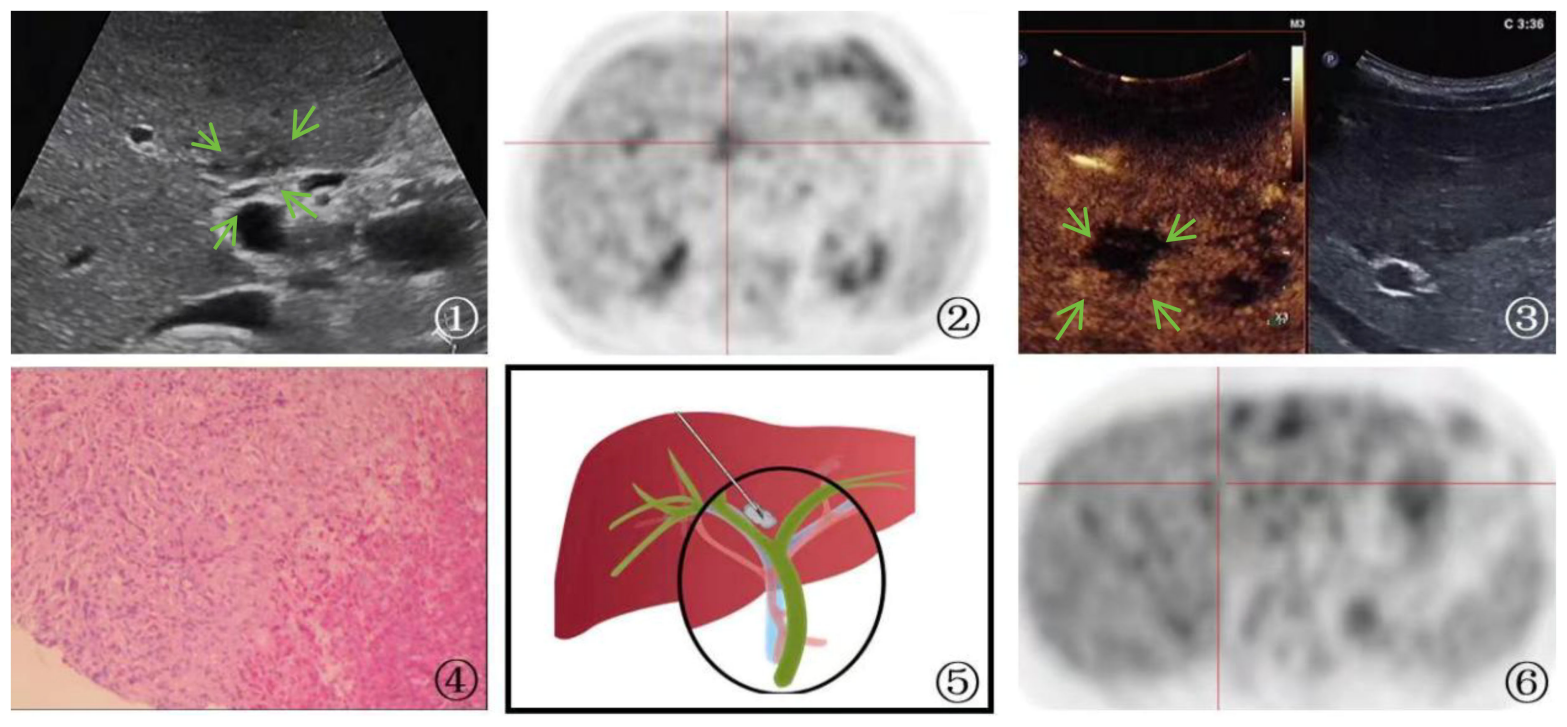
A 65-year-old man Presented with parasitic infection. ①: On ultrasound imaging, multiple patchy hypoechoic lesions(arrow) were identified surrounding the bile duct, with one particularly located at the first hepatic hilum. The boundary of the lesion is indistinct, and its irregular shape is accompanied by internal echogenicity that appears uneven. ②: PET-CT scan showed hypermetabolic activity in the first hepatic hilum lesion, indicating the possibility of tumor. ③: Ultrasound contrast demonstrated rapid wash-in and wash-out enhancement(arrow) in the first hepatic hilum lesion. ④: A schematic diagram was provided to illustrate the percutaneous biopsy procedure of the first hepatic hilum under ultrasound guidance. ⑤: High magnification microscope, HE staining(magnification, ×40);The biopsy sample of the liver demonstrated the occurrence of focal necrosis accompanied by the formation of granulomatous nodules, with a substantial infiltration of eosinophilic granulocytes and liver cell edema. The portal area displayed a chronic infiltration of inflammatory cells and fibrous tissue hyperplasia, indicating a parasitic infection. ⑥: After the therapeutic intervention, a subsequent PET-CT scan revealed a lack of metabolic elevation in the previously identified lesion located at the first hepatic hilum, indicative of its regression.

One patient who was suspected of having a primary tumor in the first hepatic hilum with multiple liver metastases underwent PUS-CNB, which diagnosed a parasitic infection. The treatment plan was then changed to internal medicine, and follow-up at 1 year showed the disappearance of the lesion (**Figure 4**).

One patient who was being evaluated for fever underwent lymph node excision biopsy in the neck, which revealed reactive proliferation on pathology. However, the treatment outcome was unsatisfactory. Subsequent PET-CT revealed a high metabolic lesion in the first hepatic hilum, and PUS-CNB confirmed it as diffuse large B-cell lymphoma. The patient was then referred to hematology for chemotherapy treatment.

One patient who was suspected of having cholangiocarcinoma based on imaging findings had a PUS-CNB that diagnosed IgG4-related sclerosing cholangitis. The treatment plan was then changed to internal medicine.

**Figure 5:**
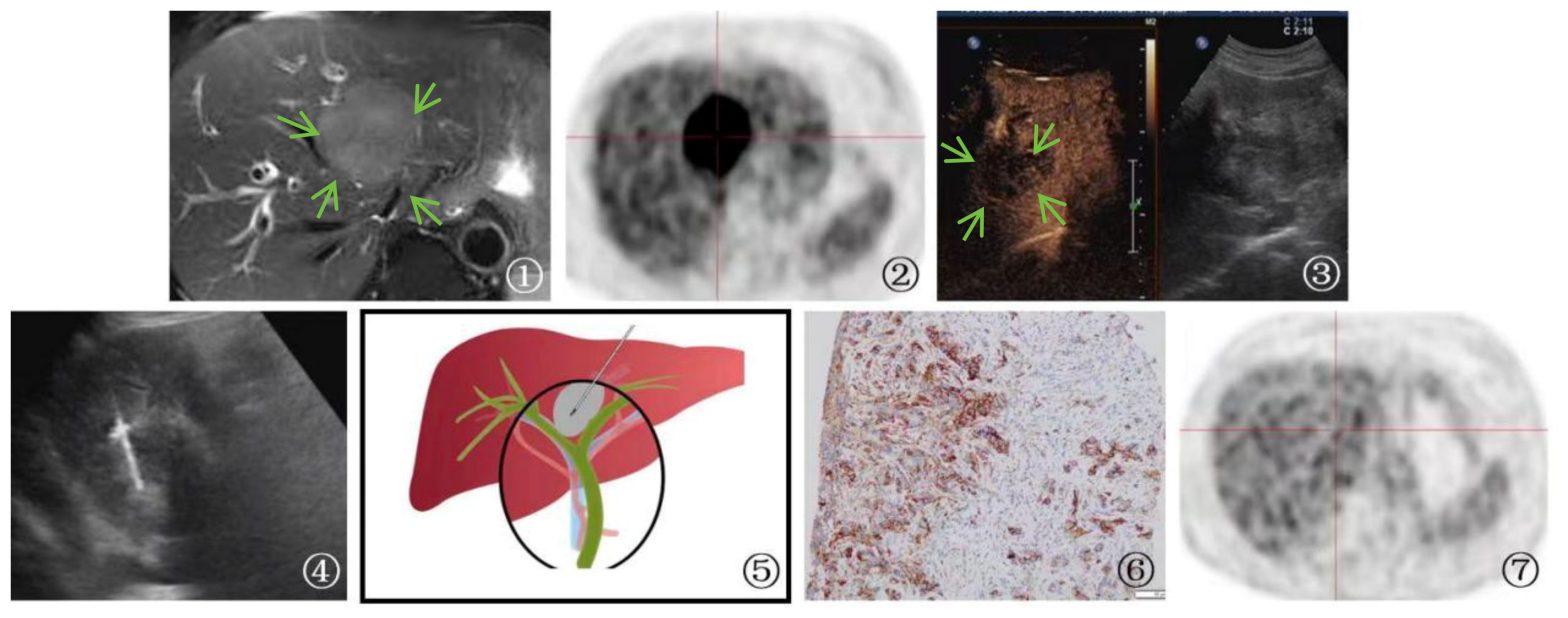
A 70-year-old male patient Presented with cholangiocarcinoma (a nodular type). ①: MRI T2-weighted magnetic resonance imaging showed a slightly elevated signal in the first hepatic hilum region(arrow), indicating a high likelihood of cholangiocarcinoma. ②: PET-CT shows high metabolic activity in the lesion, suggesting the possibility of malignant tumor. ③ An irregular hypoechoic mass(arrow) was detected in the first hepatic hilum during ultrasound examination. Further contrast-enhanced ultrasound revealed rapid wash-in and wash-out in the first hepatic hilum area, along with arterial phase enhancement. These findings suggest the presence of a tumor or lesion in the first hepatic hilum region. ④: Percutaneous biopsy of the first hepatic hilum was performed under ultrasound guidance. ⑤: A schematic diagram was provided to illustrate the percutaneous biopsy procedure of the first hepatic hilum under ultrasound guidance. ⑥: The immunohistochemical analysis for programmed death ligand 1 (PD-L1) demonstrated that the tumor cells expressed PD-L1 in a positive manner (TC+), with a positivity rate of 90%. ⑦: The imaging follow-up after immuno-oncology treatment presented a compelling outcome, revealing complete disappearance of the first hepatic hilum lesion on the PET-CT scan.

In the case of an advanced-stage cholangiocarcinoma patient, gene testing after PUS-CNB indicated high PD-L1 expression (90%). The patient received immunotherapy with Keytruda, receiving a total of 22 doses, with the first two doses at 200mg and the subsequent 20 doses at 100mg. During this period, the patient also underwent one session of radiation therapy with a dose of 500GY*6. Follow-up PET-CT showed that the lesions had mostly disappeared, with no metabolic enhancement, indicating a significant treatment response. The patient has survived for 3 years and maintains a high quality of life (**Figure 5**).

**Figure 6:**
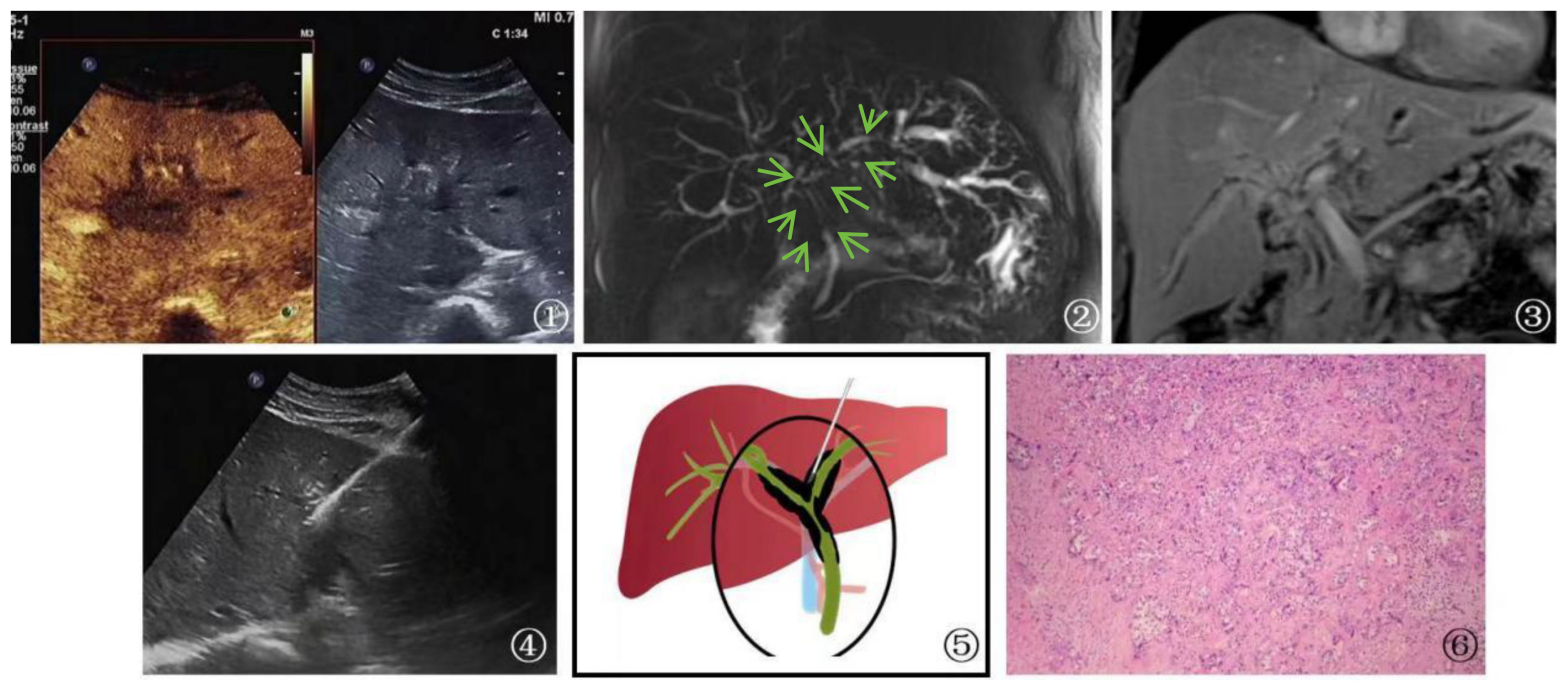
A 53-year-old female patient Presented with cholangiocarcinoma (a diffuse type). ①: According to an ultrasound examination, there is a hypoechoic lesion(arrow) with irregular margins located in the left hepatic duct at the first hepatic hilum. Additionally, a contrast-enhanced ultrasound shows that this lesion exhibits rapid wash-in and wash-out during the arterial phase. Ultrasound contrast revealed rapid wash-in and wash-out in the lesion at the first hepatic hilum. ②, ③: Cross-sectional images obtained from MRCP and MRI of the bile duct at the first hepatic hilum reveal irregular thickening of the bile duct and upper common bile duct that is surrounded by infiltrative soft tissue signals(arrow). These findings are consistent with a focal, irregular stricture in the bile duct lumen, as well as intrahepatic bile duct dilatation resembling a soft, vine-like structure with uniform and obvious enhancement. Furthermore, there is evidence of slightly longer T1 and T2 signals with uneven and obvious enhancement. Given the imaging findings, cholangiocarcinoma should be considered as a possible diagnosis. ④: Schematic diagram of percutaneous biopsy of the first hepatic hilum under ultrasound guidance. ⑤: Percutaneous biopsy of the first hepatic hilum was performed under ultrasound guidance. ⑥: High magnification microscope, HE staining, moderately differentiated cholangiocarcinoma (magnification, ×40).

Five patients with metastatic cancer underwent radiofrequency ablation treatment, and follow-up MR scans showed complete lesion inactivation in all cases. In total, 6 patients with cholangiocarcinoma, 2 patients with hepatocellular carcinoma, and 1 patient with chronic inflammation underwent surgical treatment. The remaining 24 patients had advanced-stage malignant tumors and received non-surgical treatment (**Figure 6**).

## 4 Discussion

Malignant tumors are commonly found in the first hepatic hilum, with cholangiocarcinoma, gallbladder carcinoma, metastatic carcinoma, and lymphoma being the main types. CT and MRI are widely accepted methods for further diagnosing the first hepatic hilum tumors, with MRI being the preferred imaging modality for evaluating histological characteristics and the presence of diffusion. However, even with imaging, it is not possible to completely differentiate between primary cholangiocarcinoma, metastatic carcinoma, hepatocellular carcinoma, and lymphoma (14, 15). Different tumor types require different treatment strategies, and an accurate diagnosis is crucial for clinical staging and treatment planning. Currently, the main methods of biopsy include brush cytology under ERCP, forceps biopsy, and fine-needle aspiration under endoscopic ultrasound guidance. Unfortunately, these biopsy methods are mainly focused on cytological diagnosis and have a lower sensitivity. Therefore, there is a need to explore a new, safe, and effective biopsy method.

Ultrasonography-guided percutaneous core needle biopsy (PUS-CNB) has been validated as a feasible modality for obtaining liver-peripheral tissue samples (16, 17). However, the complex anatomical structure and proximity to neighboring organs in the first hepatic hilum pose challenges in acquiring satisfactory pathological specimens. Our research has revealed that the fusion of ultrasonography with multimodal imaging cognition enables PUS-CNB to emerge as a viable technique, with a diagnostic success rate of 92.11%. In a large series of 1300 patients, accuracy rate was found as 92.8% (499/538) in total, 85% (159/187) in small needle group vs. 96.9% (340/351) in large needle group with the liver metastases (P<0.001). These rates were 91% (91/100) in total, 85.5% (47/55) in small needle group vs. 97.9% (44/45 in large needle group with hepatocellular carcinomas (P=0.039). Among 100 hepatocellular carcinomas, 18% were well-differentiated, 26% and 56% were moderate-differentiated and poor-differentiated, respectively. Biopsies of hepatocellular carcinomas were performed with large needles in 45% (45/100) and with small needles in 55% (55/100) (18).In comparison, the diagnostic success rate of endoscopic ultrasound-guided fine-needle aspiration (EUS-FNA), as reported by Krister Jones et al., stands at 32% (19). PUS-CNB significantly outperforms cytology-based biopsy methods (8–11). Moreover, our study has demonstrated the safety and efficacy of PUS-CNB for the first hepatic hilum lesions, as evidenced by the absence of major complications such as significant hemorrhage, bile leakage, intestinal perforation, infection, or needle tract seeding. In our experience, two factors have contributed to our success: Firstly, our ultrasonography interventionalists possess over a decade of expertise in procedural interventions, exhibiting a high level of proficiency and skill. Secondly, ultrasonography provides real-time dynamic visualization, facilitating the precise navigation around vital anatomical structures during the needle puncture. Efforts should be made to identify the shortest trajectory for needle insertion, avoiding critical organ structures and favoring a lateral approach along blood vessels and bile ducts. Nonetheless, there were three cases in which the intended pathological diagnosis was not successfully obtained, resulting in false negatives. In these instances, the final histopathological findings revealed cholangiocarcinoma, characterized by an abundance of fibrous tissue and chronic inflammatory cells, which may have contributed to the failure of our biopsy procedure. Insufficient tumor cell representation within the biopsy specimens can lead to both overdiagnosis and underdiagnosis. Perhaps the utilization of a larger gauge biopsy needle, such as 16G, could ameliorate this issue. Additionally, increasing the number of biopsy attempts and adopting a multidirectional approach may also serve as potential improvements. Pre-procedural ultrasonography contrast enhancement could aid in identifying suitable biopsy sites, prioritizing areas with enhanced activity while avoiding necrotic regions. These strategies may potentially enhance the overall success rate of the biopsy procedure. It needs further investigation in this setting.

In this study, 5 cases had a change in treatment plan after PUS-CNB, highlighting the important role of PUS-CNB in guiding subsequent therapies. PUS-CNB had a positive impact on diagnosis and enhanced physicians’ confidence in 97.37% of cases. Among the tumors in the first hepatic hilum, cholangiocarcinoma accounted for the majority (57.89%). Cholangiocarcinoma is a malignant tumor originating from the bile ducts and is the second most common primary malignant liver tumor after hepatocellular carcinoma, representing 10%-20% of all liver tumors (20). It has a poor prognosis, with an overall 5-year survival rate of 2%-30% (21, 22). Even with curative resection, the 5-year survival rate is less than 40% (23). Importantly, many cases of cholangiocarcinoma are diagnosed at an advanced stage, limiting the opportunity for surgical intervention. However, there are various treatment options available for advanced tumors, including biliary stent placement, percutaneous transhepatic cholangiodrainage (PTCD), chemotherapy, immunotherapy, targeted therapy, and more (24, 25). A definitive histopathological diagnosis helps guide personalized treatment approaches (26, 27). In one case of our study, a patient was diagnosed with advanced-stage cholangiocarcinoma in the first hepatic hilum based on imaging findings, precluding surgical intervention. After PUS-CNB and subsequent genetic testing, the patient was found to have high PD-L1 expression (90%), and with the combination of immunosuppressive agents and radiotherapy, the patient achieved near-complete regression of the lesion during a 3-year follow-up. Immune checkpoint inhibitors have emerged as a novel first-line treatment option for advanced cholangiocarcinoma, with multiple studies demonstrating their efficacy in this patient population (28, 29). A study by Japanese scholars further supported the reliable and well-tolerated efficacy of combination therapy using immune checkpoint inhibitors and chemotherapy compared to monotherapy with chemotherapy or immunotherapy drugs (30). Therefore, for patients with advanced-stage tumors, obtaining histopathological tissue is a crucial step in exploring non-surgical treatment options.

For lymphoma, patients can often avoid surgery due to their sensitivity to chemotherapy. Effective tumor control and long-term survival can be achieved through standardized chemotherapy, and a specific subtype can only be determined with a definite pathology to establish appropriate clinical chemotherapy protocols. In this study, in the case of diffuse large B-cell lymphoma diagnosed after PUS-CNB, obtaining tissue specimens was crucial. For patients with metastatic cancer, radiofrequency ablation (RFA) serves as a palliative treatment modality. The percutaneous RFA procedure for lesions in the first hepatic hilum under ultrasound guidance is similar to PUS-CNB, making ultrasound-guided RFA feasible for lesions in the first hepatic hilum. In this study, there were 5 patients who were diagnosed with metastatic cancer after PUS-CNB confirmed the first hepatic hilum lesions. All of them underwent ultrasound-guided RFA for the first hepatic hilum lesions, and during a follow-up period of six months, the lesions were in an inactive state. Fusion imaging can also reduce false-positive lesion detection during US-guided RFA and consistently improve the detection of HCCs, especially when these are smaller than 2 cm. The ability of fusion imaging to reduce false positives also applies to the evaluation of local tumor progression after RFA and TACE (31, 32).

In this study, the integration of ultrasound with multimodal image cognition in percutaneous ultrasound-guided the first hepatic hilum puncture biopsy offers the following advantages:1.High safety: Prior to the procedure, other imaging examinations are performed, and with the integration of ultrasound cognition, large blood vessels and bile ducts can be avoided. The appropriate puncture path can be selected, reducing the risk of major bleeding and bile leakage. This study found that punctures above the common hepatic duct in the first hepatic hilum can be performed through the intercostal approach, while lesions in the upper segment of the common bile duct and the surrounding hepatic hilum can be accessed through the subcostal approach, reducing the puncture distance. Real-time dynamic monitoring of the needle insertion process ensures a safe and controlled procedure. Additionally, multiplane imaging provides confidence for operators.2.Simplicity, speed, and ease of use: This technique is easy to learn, and experienced ultrasound interventionists can quickly master it after short training. The puncture biopsy procedure is relatively short, with a minimum time of 9 minutes and an average time of 14.55 ± 2.73 minutes.3.High accuracy: The use of an 18-gauge thick needle allows for an adequate amount of pathological tissue to be obtained, resulting in higher diagnostic accuracy compared to previous fine needle aspiration biopsy methods.4.Ultrasound examination features no radiation, is flexible, and has low cost: Compared to fluoroscopy or CT-guided puncture biopsy, ultrasound’s radiation-free imaging is an absolute advantage. Moreover, the overall procedure incurs minimal economic costs.

However, there are certain limitations to consider. Firstly, this procedure is not without risks. Despite the integration with other imaging modalities, potential complications such as vascular injury, bile leakage, and needle tract seeding still exist. It also relies on the experience and radiological expertise of the operator. Post-procedure observation for one hour and close collaboration with multiple clinical disciplines are necessary to promptly identify and manage any complications that may arise. Secondly, this study is retrospective and has a relatively small number of cases. Patients without a puncture path were not included in the study, which may have inflated the success rate of the procedure. Additionally, the study lacks a comparison with other imaging-guided techniques. Retrospective data collected from a single institution might introduce bias. Therefore, large-scale, high-quality prospective studies are needed to validate these findings and obtain more accurate conclusions.

## 5 Conclusion

The integration of ultrasound and multimodal imaging cognition in the percutaneous ultrasound-guided biopsy of the first hepatic hilum lesions can reduce unnecessary surgical exploration or resection. It can confirm the resectability or treatability of the lesions and has the potential to become a new, superior, and faster method. This approach can optimize the triage and treatment strategies for indeterminate hepatic hilum lesions, benefiting the patients. It is particularly valuable for patients with unclear lesions in the first hepatic hilum who require a definitive diagnosis, as well as for those with bile duct carcinoma who have lost the opportunity for surgical intervention and can benefit from immunotherapy.

## Data availability statement

The raw data supporting the conclusions of this article will be made available by the authors, without undue reservation.

## Author contributions

X-TZ: Supervision, Writing – original draft, Writing – review & editing. XL: Data curation, Writing – original draft, Writing – review & editing. Z-LH: Conceptualization, Investigation, Writing – original draft, Writing – review & editing. SC: Visualization, Writing – original draft. J-CY: Investigation, Writing – original draft. Y-cL: Supervision, Writing – original draft. S-SW: Conceptualization, Writing – original draft, Writing – review & editing.
